# Estimated Cardiorespiratory Fitness and Incident Cardiometabolic Multimorbidity in Older Adults: A Prospective Cohort Study

**DOI:** 10.1016/j.mayocpiqo.2026.100741

**Published:** 2026-07-27

**Authors:** Setor K. Kunutsor, Reyhaneh Rikhtehgaran, Sae Young Jae, Jari A. Laukkanen

**Affiliations:** a Section of Cardiology, Department of Internal Medicine, Max Rady College of Medicine, Rady Faculty of Health Sciences, University of Manitoba, Canada; b Division of Global, Lifestyle and Metabolic Health, Leicester Real World Evidence Unit, Diabetes Research Centre, University of Leicester, Leicester General Hospital, Leicester, UK; c Department of Sport Science, University of Seoul, Republic of Korea; d Institute of Clinical Medicine, Department of Medicine, University of Eastern Finland, Kuopio, Finland; e Wellbeing Services County of Central Finland, Department of Medicine, Jyväskylä, Finland

## Abstract

**Objective:**

To evaluate the association of estimated cardiorespiratory fitness (eCRF) with incident cardiometabolic multimorbidity (CMM) and its potential contribution to CMM risk prediction in an older adult population.

**Patients and Methods:**

The analysis included 3326 participants from the English Longitudinal Study of Aging (mean age 63 years; 1820 (54.7%) women) who were free of hypertension, cardiovascular disease, diabetes, and stroke at baseline (wave 4; 2008-2009). Estimated CRF was derived using validated nonexercise prediction equations (heart rate-based, body mass index-based, and Nord-Trøndelag Health Study-based eCRF). Incident CMM was defined at wave 10 (2021-2023) as the presence of ≥2 of the following: hypertension, cardiovascular disease, diabetes, or stroke. Multivariable-adjusted odds ratios (ORs) (95% CIs) and discrimination indices were calculated.

**Results:**

During 12-15 years of follow-up, 197 (5.9%) participants developed CMM. Each eCRF measure reported an inverse linear association with CMM risk. Higher heart rate-based eCRF was associated with lower odds of CMM (per 1- metabolic equivalents (MET) increment: OR=0.78; 95% CI, 0.70-0.87; highest vs lowest tertile: OR=0.42; 95% CI, 0.25-0.69). Similar associations were observed for body mass index-based eCRF (per 1-MET increment: OR=0.78; 95% CI, 0.68-0.89; highest vs lowest tertile: OR=0.42; 95% CI, 0.25-0.70) and Nord-Trøndelag Health Study-based eCRF (per 1-MET increment: OR=0.73; 95% CI, 0.65-0.82; highest vs lowest tertile: OR=0.36; 95% CI, 0.22-0.58). Incorporation of each eCRF measure into a conventional risk prediction model modestly improved discrimination.

**Conclusion:**

Estimated CRF derived from validated nonexercise equations was similarly and inversely associated with incident CMM in older adults, with all models reporting comparable modest predictive utility.

Cardiometabolic multimorbidity (CMM), commonly defined as the co-occurrence of 2 or more cardiometabolic conditions such as hypertension, type 2 diabetes, stroke, and cardiovascular disease (CVD), is an increasingly prevalent global health challenge.^1^^,^^2^ Individuals with CMM experience markedly poorer health outcomes and higher mortality risk compared with those with single cardiometabolic conditions.^3^^,^^4^ Although established risk factors, including older age, adiposity, unhealthy diet, smoking, socioeconomic disadvantage, and physical inactivity, contribute to the development of CMM,^5^ considerable residual risk remains, suggesting the potential role of unexplored factors.

Cardiorespiratory fitness (CRF), an integrative measure of the capacity of the circulatory, respiratory, and muscular systems to deliver and utilize oxygen during physical exertion, is an objective physiological indicator of functional health and exercise capacity.^6^ Although CRF is influenced by age, sex, genetics, and underlying health status, habitual physical activity (PA) and structured exercise training remain the most important modifiable determinants.^7^ Higher levels of CRF have been consistently associated with lower risks of hypertension, type 2 diabetes, CVD, stroke, and all-cause mortality,^8^, ^9^, ^10^, ^11^, ^12^, ^13^ leading the American Heart Association to advocate for the routine consideration of CRF as a clinical vital sign.^14^

The gold standard measure of CRF is the direct assessment of maximal oxygen uptake during cardiopulmonary exercise testing; however, such assessments are often impractical in large-scale epidemiological studies because they require specialized equipment, trained personnel, maximal participant effort, and considerable time and financial resources. Consequently, nonexercise prediction equations have emerged as practical alternatives for estimating CRF in population-based studies.^15^ Several eCRF models have been developed, including the widely used Jackson equations^21^^,^^22^ and the Nord-Trøndelag Health Study (HUNT) equations developed by Nes et al,^16^ which were derived from directly measured peak oxygen uptake (VO_2_peak) in a large healthy population and subsequently validated as predictors of long-term cardiovascular and all-cause mortality.^17^ Although eCRF does not replace objectively measured CRF and is subject to prediction error, accumulating evidence suggests that it captures important physiological information beyond self-reported PA and serves as a robust marker of future adverse health outcomes.^18^^,^^19^

Despite the established protective role of PA against CMM and the growing recognition of CRF as an important cardiometabolic health marker, evidence evaluating the association between CRF and the risk of CMM remains limited. To date, studies examining PA and CMM have substantially outnumbered those evaluating CRF,^20^^,^^21^ and our review of the literature identified only one prior study assessing the relationship between CRF and CMM, which was conducted in the UK Biobank.^22^ Further evidence from independent cohorts is needed to validate and extend these findings, particularly in older adults who are at heightened risk of developing multimorbidity. In addition, the potential utility of eCRF in improving prediction of future CMM risk has not been evaluated. Therefore, using data from the English Longitudinal Study of Aging (ELSA), we aimed to assess the nature and magnitude of the association between eCRF and incident CMM in older adults and to evaluate the predictive utility of eCRF for CMM using two established nonexercise CRF prediction equations (heart rate- and body mass index (BMI)-based) developed by Jackson et al,^23^^,^^24^ which were selected because of their widespread use and validation in epidemiological studies of cardiometabolic and mortality outcomes. As a subsidiary aim, we further evaluated the robustness of the findings using the sex-specific nonexercise eCRF equations developed by Nes et al^16^ from the HUNT as a sensitivity analysis (henceforth referred to as HUNT-based eCRF).

## Patients and Methods

### Study Population

This investigation was reported in accordance with the Strengthening the Reporting of Observational Studies in Epidemiology recommendations (Supplemental Appendix 1, available online at http://www.mcpiqojournal.org).^25^ Data were drawn from the ELSA, an ongoing population-based cohort of adults aged 50 years and older living in private households across England.^26^ ELSA was established using participants recruited from the Health Survey for England conducted in 1998, 1999, and 2001, with the first ELSA wave undertaken in 2002-2003 and including 11,391 individuals aged ≥50 years. Participants have subsequently undergone repeat assessments approximately every 2 years.^26^ For the present analysis, wave 4 (2008-2009) was treated as baseline, with participants followed through wave 10 (2021-2023). To evaluate incident CMM, we excluded individuals with prevalent hypertension, CVD, diabetes, or stroke at baseline, thereby restricting the cohort to participants initially free of major cardiometabolic disease.^27^, ^28^, ^29^ We further excluded participants with missing information on variables required for eCRF derivation, covariates, or CMM ascertainment. Following these exclusions, 3326 participants were included in the final analytic sample using a complete-case approach (Supplemental Appendix 2, available online at http://www.mcpiqojournal.org). Written informed consent was obtained from all participants, and ethical approval for ELSA was granted by the London Multicenter Research Ethics Committee.

### Assessments of Covariates and Outcome

Information on sociodemographic, behavioral, and health-related factors was obtained through nurse-administered structured interviews and standardized self-completed questionnaires. These data collection methods, which have been extensively applied in prior ELSA investigations, provided information on age, sex, smoking status, alcohol intake, PA, and medical history.^26^^,^^30^, ^31^, ^32^ Alcohol use was determined from participant reports of drinking frequency over the previous 12 months. Smoking status was assessed using a sequential approach whereby participants first indicated whether they had ever smoked cigarettes; those responding affirmatively were then asked whether they were current smokers at the time of assessment.^31^ Habitual PA was evaluated using a validated questionnaire capturing participation in light-, moderate-, and vigorous-intensity activities. Consistent with standard ELSA procedures, participants were categorized as sedentary (no moderate or vigorous activity), low (light activity only), moderate (moderate activity at least once weekly), or high (vigorous activity at least once weekly).^26^^,^^30^ Participants also attended standardized nurse-led clinical assessments at Mobile Examination Centers, during which anthropometric, physiological, and functional measurements were collected together with venous blood samples for biomarker analyses. Height, body weight, waist circumference (WC), handgrip strength (HGS), and blood pressure were measured according to standardized protocols. Body weight was recorded to the nearest 0.1 kg using calibrated electronic scales with participants barefoot and wearing light clothing, and WC was measured to the nearest even millimeter midway between the lower rib margin and iliac crest.^33^ Body mass index (BMI) was calculated as weight divided by height squared (kg/m^2^). Handgrip strength was assessed using a Smedley dynamometer (Stoelting Co). Participants completed 3 trials with each hand in alternating sequence beginning with the dominant hand, with 1-minute rest intervals between attempts; the highest value achieved across all 6 trials was used in analyses.^34^ Blood pressure and resting heart rate were measured using an automated Omron HEM-907 monitor (OMRON Healthcare Europe BV). Three seated readings were obtained from the right arm, and the average of the second and third measurements was used for analysis.^33^ Prevalent chronic conditions, including hypertension, CVD, diabetes, and stroke, were identified from self-reported physician diagnoses.^31^ Incident CMM was defined at wave 10 as the presence of at least two of the following conditions: hypertension, CVD, diabetes, or stroke.^27^, ^28^, ^29^^,^^32^

### Assessment of eCRF

Heart rate-based eCRF was calculated using sex-specific nonexercise prediction equations derived from the Aerobics Center Longitudinal Study by Jackson et al.^24^ These equations incorporate age, resting heart rate (RHR), WC, BMI, smoking status, and PA:

Men: eCRF (METs)=21.2870 + (0.1654 × Age) − (0.0023 × Age^2^) − (0.2318 × BMI) − (0.0337 × WC) − (0.0390 × RHR) + (0.6351 × PA) − (0.4263 × Smoking)

Women: eCRF (METs)=14.7873 + (0.1159 × Age) − (0.0017 × Age^2^) − (0.1534 × BMI) − (0.0085 × WC) − (0.0364 × RHR) + (0.5987 × PA) − (0.2994 × Smoking)

BMI-based eCRF was estimated using the validated sex-specific nonexercise prediction equation originally proposed by Jackson et al, which incorporates age, BMI, sex, and habitual PA rating (PA-R):^23^

BMI-based eCRF=50.513 + (1.589 × PA-R) − (0.289 × Age) − (0.552 × BMI) + (5.863 × Sex)

Because the original Jackson 0-7 PA-R scale was unavailable in ELSA, ELSA PA categories were converted to approximate PA-R scores based on increasing activity level as follows: sedentary=0, low=2, moderate=4, and high=6. This mapping was used to approximate the Jackson PA-R scale while preserving the ordinal gradient of habitual activity. The BMI-based equation yields CRF estimates in mL/kg/min, which were converted to metabolic equivalents (METs) by dividing by 3.5.

The HUNT sex-specific nonexercise prediction equation developed by Nes et al^16^ incorporates age, WC, heart rate, sex, and a PA summary index (PA-I):

Men: 100.27 − (0.296 × age) − (0.369 × WC) − (0.155 × RHR) + (0.226 × PA-I)

Women: 74.74 − (0.247 × age) − (0.259 × WC) − (0.114 × RHR) + (0.198 × PA-I)

Since the PA-I used in the original HUNT nonexercise eCRF equations was unavailable in ELSA, an approximate PA-I was derived by mapping ELSA PA categories according to increasing activity levels (sedentary=0, low=1, moderate=5, and high=10). This recoding was undertaken to provide the closest approximation to the HUNT PA-I while maintaining the ordinal gradient of habitual PA. The HUNT equation yields CRF estimates in mL/kg/min, which were converted to METs by dividing by 3.5.

### Statistical Analyses

Participant characteristics at baseline were described using standard summary statistics. Continuous variables are presented as mean (SD) or median with interquartile range (IQR), according to data distribution, whereas categorical variables are expressed as counts and percentages. Comparisons between groups were performed using independent-samples t tests for continuous measures and χ^2^ tests for categorical variables. Dose-response associations between eCRF measures and incident CMM were examined using natural cubic spline regression models. Splines were specified with knots placed at the 10th, 50th, and 90th percentiles of the eCRF distribution. For graphical presentation, continuous covariates were held at their median values and categorical covariates at their modal categories. Predicted dose-response curves were plotted across the 1st to 99th percentiles of eCRF to minimize the influence of extreme values. Multivariable logistic regression models were used to estimate odds ratios (ORs) and 95% CIs for the associations. Two adjustment models were used to account for potential confounding - Model 1: adjusted for age and sex and Model 2: Model 1 plus alcohol consumption, systolic blood pressure (SBP), total cholesterol, high-density lipoprotein cholesterol (HDL-C), triglycerides (TGs), and HGS. Covariates were chosen on the basis of their known relationships with cardiometabolic disease, prior findings from analyses conducted in the ELSA cohort,^27^, ^28^, ^29^^,^^32^^,^^35^ and their likely potential to confound the associations of interest.^36^ Although age and sex are components of the eCRF prediction equations, models were adjusted for age and sex because these are strong determinants of CMM and may confound the association between eCRF and incident CMM. Variables directly included in the eCRF equations were not additionally adjusted for in the main models to avoid overadjustment. The exposures were modeled per 1-MET increment and as tertiles. The largely linear dose-response relationships observed between eCRF measures and CMM suggest that modeling eCRF as a continuous measure may be the most appropriate approach for characterizing its association with CMM risk and maximizing statistical efficiency. Tertile analyses were performed as a secondary approach to facilitate interpretation of risk gradients. Tertiles were selected because of the limited number of incidents CMM events. Stata MP version 18.0 (StataCorp LLC) and R (version 4.0.4, R Foundation for Statistical Computing) were used for statistical analyses.

## Results

### Baseline Characteristics

The overall baseline characteristics of the study population and according to incident CMM status are summarized in Table 1. Among the 3326 participants included in the analysis, the mean ± SD age at baseline was 63.4 ± 8.4 years, and 1820 (54.7%) were women. The mean ± SD heart rate-based, BMI-based CRF, and HUNT-based were 9.22, 7.47, and 9.14 METs, respectively. Individuals who developed CMM during follow-up exhibited lower baseline levels of all eCRF measures and higher levels of anthropometric measures (BMI and WC) and heart rate. They were also more frequently current smokers and exhibited higher SBP together with a more adverse lipid profile and reflected by increased TG levels and lower HDL-C concentrations.

**Table 1:** Baseline Characteristics Overall and by Cardiometabolic Multimorbidity Status

Characteristic	Overall (N=3326)	No CMM (N=3129)	Yes CMM (N=197)	*P*
	Mean ± SD, Median (IQR), or n (%)	Mean ± SD, Median (IQR), or n (%)	Mean ± SD, Median (IQR), or n (%)	
Heart rate-based eCRF, METs	9.22 ± 1.94	9.25 ± 1.94	8.78 ± 1.86	.001
BMI-based eCRF, METs	7.47 ± 1.53	7.49 ± 1.53	7.18 ± 1.54	.006
HUNT-based eCRF, METs	9.14 ± 1.64	9.17 ± 1.63	8.61 ± 1.69	<.001
Waist circumference, cm	94.9 ± 13.2	94.4 ± 12.9	101.8 ± 15.2	<.001
BMI, kg/m^2^	27.5 ± 4.7	27.3 ± 4.6	29.8 ± 5.6	<.001
Heart rate (bpm)	66.6 ± 10.2	66.5 ± 10.1	68.9 ± 10.5	.001
Age (y)	63.4 ± 8.4	63.4 ± 8.5	62.6 ± 6.5	.17
Sex				.11
Female	1820 (54.7%)	1723 (55.1%)	97 (49.2%)	
Male	1506 (45.3%)	1406 (44.9%)	100 (50.8%)	
Ethnicity				.75
White	3266 (98.3%)	3073 (98.3%)	193 (98.0%)	
Non-white	58 (1.7%)	54 (1.7%)	4 (2.0%)	
Current smoker				.037
No	2852 (85.7%)	2693 (86.1%)	159 (80.7%)	
Yes	474 (14.3%)	436 (13.9%)	38 (19.3%)	
Alcohol categories				.83
None	1106 (33.3%)	1036 (33.1%)	70 (35.5%)	
1-2 times/wk	839 (25.2%)	793 (25.3%)	46 (23.4%)	
3-4 times/wk	635 (19.1%)	600 (19.2%)	35 (17.8%)	
5 or more times/wk	746 (22.4%)	700 (22.4%)	46 (23.4%)	
Handgrip strength, kg	31.0 (11.4)	30.9 (11.3)	32.7 (11.9)	.033
Systolic blood pressure, mm Hg	130 (17)	130 (17)	137 (17)	<.001
Total cholesterol, mmol/L	5.84 (1.14)	5.86 (1.13)	5.59 (1.20)	.002
HDL cholesterol, mmol/L	1.60 (0.42)	1.60 (0.42)	1.45 (0.41)	<.001
Triglycerides, mmol/L	1.40 (1.00-2.00)	1.40 (1.00-2.00)	1.70 (1.20-2.50)	<.001
PA level				.14
Sedentary	84 (2.5%)	78 (2.5%)	6 (3.0%)	
Low	581 (17.5%)	535 (17.1%)	46 (23.4%)	
Moderate	1807 (54.3%)	1707 (54.6%)	100 (50.8%)	
High	854 (25.7%)	809 (25.9%)	45 (22.8%)	

Abbreviations: BMI, body mass index; CMM, cardiometabolic multimorbidity; eCRF, estimated cardiorespiratory fitness; HDL, high-density lipoprotein; HUNT, Nord-Trøndelag Health Study; IQR, interquartile range; METs, metabolic equivalents; PA, physical activity; SD, standard deviation.

### Associations of eCRF Measures With CMM

**Figure 1:**
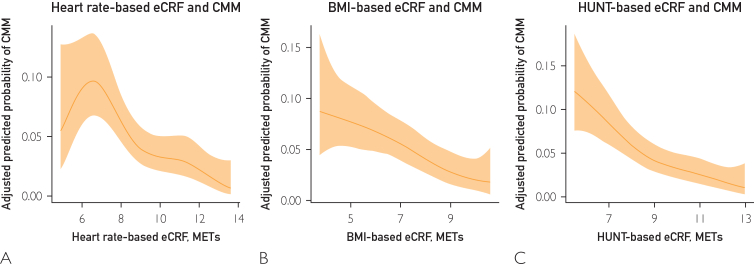
Spline curves of the associations of eCRF measures with the risk of cardiometabolic multimorbidity. (A) Heart rate-based eCRF, (B) BMI-based eCRF, and (C) HUNT-based eCRF. BMI, body mass index; eCRF, estimated cardiorespiratory fitness; HUNT, Nord-Trøndelag Health Study. The model was adjusted for age, sex, alcohol consumption, systolic blood pressure, total cholesterol, high-density lipoprotein cholesterol, triglycerides, and handgrip strength.

**Figure 2:**
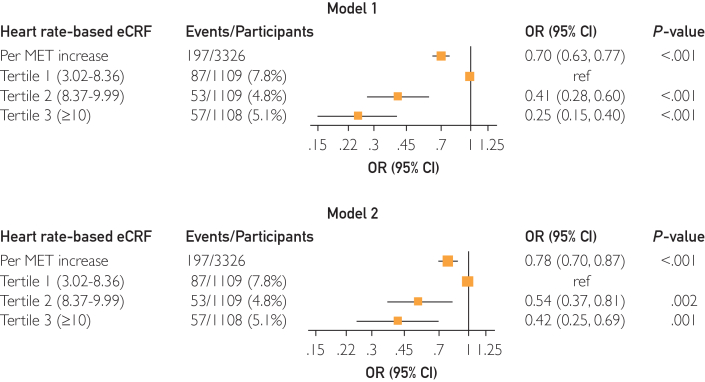
Associations of heart rate-based estimated cardiorespiratory fitness with cardiometabolic multimorbidity. OR, odds ratio; Values in brackets represent the absolute event proportions. Model 1: Adjusted for age and sex. Model 2: Model 1 plus alcohol consumption, systolic blood pressure, total cholesterol, high-density lipoprotein cholesterol, triglycerides, and handgrip strength.

Over a follow-up duration of 12-15 years, 197 (5.9%) cases of CMM were recorded. Figure 1 illustrates the dose-response relationships between eCRF measures and incident CMM. For heart rate-based eCRF, the spline curve suggested a broadly inverse association with CMM risk across the observed range of fitness values, although some nonlinearity was evident at lower eCRF levels, with risk peaking at approximately 6-7 METs before declining progressively at higher fitness levels (Figure 1A). In analysis adjusted for age and sex, each 1-MET increment in heart rate-based eCRF was associated with a lower odds of CMM (OR=0.70; 95% CI, 0.63-0.77) (Figure 2, Model 1). This association was minimally attenuated on further adjustment for alcohol consumption, SBP, total cholesterol, HDL-C, TG, and HGS (OR=0.78; 95% CI, 0.70-0.87) (Figure 2, Model 2). In tertile analyses, the corresponding estimates were OR=0.25; 95% CI, 0.15-0.40, and OR=0.42; 95% CI, 0.25-0.69 (Figure 2).

**Figure 3:**
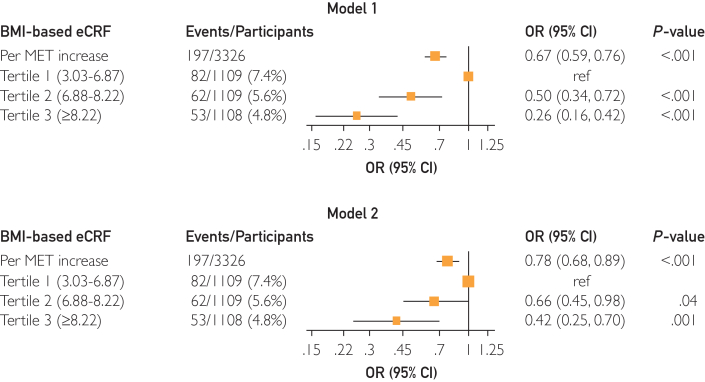
Associations of body mass index-based estimated cardiorespiratory fitness with cardiometabolic multimorbidity. OR, odds ratio; Values in brackets represent the absolute event proportions. Model 1: Adjusted for age and sex. Model 2: Model 1 plus alcohol consumption, systolic blood pressure, total cholesterol, high-density lipoprotein cholesterol, triglycerides, and handgrip strength.

BMI-based eCRF reported a more consistent monotonic inverse relationship with CMM risk, with progressively lower predicted probability of CMM observed across increasing eCRF levels (Figure 1B). In age-adjusted and sex-adjusted analysis, each 1-MET increment in the BMI-based eCRF was associated with lower odds of CMM (OR=0.67; 95% CI, 0.59-0.76) (Figure 3, Model 1). The association was slightly attenuated following alcohol consumption, SBP, total cholesterol, HDL-C, TG, and HGS (OR=0.78; 95% CI, 0.68-0.89) (Figure 3, Model 2). Comparing the highest vs lowest tertile of BMI-based eCRF, the corresponding adjusted estimates were (Model 1: OR=0.26; 95% CI, 0.16-0.42 and Model 2: OR=0.42; 95% CI, 0.25-0.70) (Figure 3).

The HUNT-based eCRF showed an inverse, approximately linear association with CMM risk, with progressively lower predicted risk observed at higher fitness levels (Figure 1C). In age-adjusted and sex-adjusted analyses, each 1-MET increment in HUNT-based eCRF was associated with a lower odds of CMM (OR=0.66; 95% CI, 0.59-0.73) (Supplementary Material 3). The association was modestly attenuated but remained statistically significant after further adjustment for alcohol consumption, SBP, total cholesterol, HDL-C, TG, and HGS (OR=0.73; 95% CI, 0.65-0.82) (Supplementary Material 3). When comparing participants in the highest vs lowest tertile of HUNT-based eCRF, the corresponding estimates were OR=0.23 (95% CI, 0.15-0.36) in the age-adjusted and sex-adjusted model and OR=0.36 (95% CI, 0.22-0.58) after multivariable adjustment (Supplemental Appendix 3, available online at http://www.mcpiqojournal.org).

### Risk Prediction

Table 2 presents the findings from the risk prediction analyses. The conventional risk factor model reported a C-index of 0.6837 (95% CI, 0.6439-0.7236). Incorporation of heart rate-based eCRF increased the C-index to 0.6959 (95% CI, 0.6565-0.7353), representing a modest but nonsignificant improvement in discrimination (Δ=0.0122, *P*=.14). Nevertheless, the addition of heart rate-based eCRF was associated with a significant enhancement in model fit according to the −2 log likelihood test (*P*<.001).

**Table 2:** Measures of Risk Discrimination On Addition of eCRF Measures to a CMM Risk Model Containing Established Risk Factors

Measure of discrimination	Heart rate Based-eCRF	BMI-based eCRF	HUNT-based eCRF
C-index (95% CI): conventional risk factors	0.6837 (0.6439-0.7236)	0.6837 (0.6439-0.7236)	0.6837 (0.6439-0.7236)
C-index (95% CI): conventional risk factors plus exposure	0.6959 (0.6565-0.7353)	0.6926 (0.6527-0.7326)	0.6999 (0.6601-0.7396)
C-index change (*P* value)	0.0122 (.14)	0.0089 (.24)	0.0162 (.10)
*P* value for difference in −2 log likelihood	<.001	<.001	<.001

Conventional risk factors include age, sex, alcohol consumption, systolic blood pressure, total cholesterol, high-density lipoprotein cholesterol, triglycerides, and handgrip strength.

Abbreviations: BMI, body mass index; CMM, cardiometabolic multimorbidity; eCRF, estimated cardiorespiratory fitness; HUNT, Nord-Trøndelag Health Study.

Similarly, inclusion of BMI-based eCRF yielded a small, nonsignificant increase in the C-index (Δ = 0.0089, *P*=.24) and likewise significantly improved model fit (*P*<.001). Qualitatively similar findings were observed for HUNT-based eCRF (Table 2).

## Discussion

In this prospective cohort of older adults from England followed for up to 15 years, participants who developed CMM exhibited a less favorable baseline cardiometabolic profile, including lower levels of eCRF, compared with those who remained free of CMM. Dose-response analyses reported broadly inverse associations between eCRF measures and subsequent CMM risk, with little evidence of nonlinearity, suggesting a graded reduction in risk across increasing eCRF levels. Higher heart rate-, BMI-, and HUNT-based eCRF were each independently associated with lower odds of incident CMM after adjustment for established cardiometabolic risk factors, and the magnitude of these associations was similar across all eCRF metrics. In addition, incorporation of each eCRF measure into a conventional risk prediction model resulted in similarly modest improvements in discrimination and model fit, with no eCRF approach reporting a clear advantage in predictive performance.

Despite the extensive literature linking CRF—measured using both objective and estimated approaches—to adverse cardiovascular and metabolic outcomes,^8^, ^9^, ^10^, ^11^, ^12^, ^13^ evidence specifically relating CRF to CMM remains limited. To our knowledge, only one previous study has directly examined this association. In a large UK Biobank analysis of 47,484 adults free of cardiometabolic disease at baseline, Chen and colleagues reported that higher CRF, assessed using a submaximal cycle ergometer test, was associated with a lower risk of progression from a healthy state to first cardiometabolic disease and subsequently to CMM.^22^ Our findings are consistent with and extend this prior work by reporting that validated nonexercise estimated CRF measures are similarly associated with lower long-term risk of CMM, suggesting that eCRF may offer a practical alternative for multimorbidity risk stratification in older adults when direct fitness assessment is not feasible.

These findings have several potential clinical and public health implications. First, because eCRF can be derived from simple, routinely collected variables, it may provide a pragmatic alternative for multimorbidity risk stratification in settings where direct fitness testing is not feasible. The observed graded and independent associations between these eCRF measures and CMM support their potential value as markers of early cardiometabolic vulnerability beyond conventional demographic, lifestyle, and clinical risk factors. Although the addition of eCRF measures to models containing conventional risk factors significantly improved overall model fit, as indicated by the −2 log likelihood test, improvements in discrimination were small and not statistically significant. These findings suggest that eCRF may capture additional information related to CMM risk beyond established risk factors; however, the magnitude of improvement in identifying individuals who will or will not develop CMM appears limited. However, the low cost, accessibility, and ease of implementation of these measures support consideration of eCRF within clinical and public health screening frameworks. This may be particularly relevant given that, despite recommendations from the American Heart Association to consider CRF as a clinical vital sign,^14^ objective CRF assessment remains infrequently performed in routine practice. Although eCRF may be useful as an adjunctive marker within broader cardiometabolic risk assessment strategies, the clinical relevance of its incremental predictive value requires further evaluation in larger studies and diverse populations.

This study has several important strengths. To the best of our knowledge, it is among the earliest prospective investigations to examine the association between eCRF and incident CMM and the first to evaluate whether eCRF improves CMM risk prediction beyond conventional risk factors. The study was conducted in a large, nationally representative cohort of older adults with long-term follow-up, providing important evidence in a population at particularly high risk of multimorbidity. A further strength is the use of multiple nonexercise eCRF prediction models, including the Jackson and HUNT equations, which reported consistent findings across different approaches to eCRF estimation. Additionally, we performed comprehensive analyses incorporating adjustment for a broad range of established and emerging cardiometabolic risk factors and assessed the shapes of the dose-response relationships between eCRF and CMM. Several limitations should also be acknowledged. First, CRF was estimated using nonexercise prediction equations rather than directly measured using objective assessments such as VO_2_peak. Although these equations provide a practical and scalable approach for estimating CRF in large population studies, they are subject to inherent limitations, including their inability to capture genetic influences on CRF, reliance on self-reported inputs such as PA, and potential overestimation or underestimation of CRF at the extremes of the fitness distribution.^14^^,^^37^^,^^38^ In addition, the eCRF equations were not specifically developed or calibrated for the ELSA population, and the absence of directly measured CRF data prevented assessment of their agreement with objectively measured fitness in this cohort. Nevertheless, the aim of this study was not to validate or recalibrate existing eCRF equations but to evaluate whether established eCRF metrics derived from widely used prediction models were associated with future CMM risk. The consistent findings observed across multiple eCRF equations, including the Jackson and HUNT models, support the robustness of the observed associations. A second limitation relates to the derivation of PA inputs required for the nonexercise eCRF equations. Because the original Jackson PA rating (PA-R) and HUNT PA index (PA-I) were not available in ELSA, ELSA PA categories were mapped to approximate scores based on increasing levels of habitual activity. Although this approach allowed application of established eCRF models, differences in the content, frequency, intensity, and scaling of the original and adapted PA measures may have introduced exposure misclassification and affected the accuracy of eCRF estimation. Nevertheless, the consistency of findings across multiple eCRF equations supports the overall robustness of the observed associations. Third, the observational design precludes definitive causal inference, and residual confounding from unmeasured or imperfectly measured variables remains possible. Fourth, several covariates and the CMM outcome were based primarily on self-reported data, including physician-diagnosed chronic conditions, which may introduce recall error, reporting bias, and outcome misclassification. Although prior validation work has reported moderate concordance between self-reported and clinically verified diagnoses,^39^^,^^40^ some misclassification is unavoidable. We also cannot exclude the possibility that some participants had undiagnosed cardiometabolic disease at baseline despite exclusion of those with known diagnoses. Fifth, the inclusion of hypertension within the CMM definition may have contributed substantially to the observed outcome burden, given that hypertension is common and often precedes other cardiometabolic diseases. Nevertheless, this approach is consistent with most previous CMM studies and reflects the broader continuum of cardiometabolic disease progression. A more restrictive CMM definition excluding hypertension resulted in a very small number of events in our cohort, precluding meaningful sensitivity analyses. Sixth, the absence of precise dates of disease onset precluded time-to-event analyses and necessitated the use of logistic regression-based approaches. Seventh, the relatively small number of incident CMM cases limited statistical power for detailed subgroup analyses among relevant subgroups such as age group, sex, obesity status, PA level, or baseline cardiometabolic risk. Finally, because the cohort comprised older adults living in England, the findings may not be fully generalizable to younger individuals or populations with different ethnic or geographic backgrounds. These limitations should be considered when interpreting our findings and underscore the need for further research.

## Conclusion

In this nationally representative cohort of older English adults, higher eCRF was consistently associated with a lower long-term risk of CMM in a graded dose-response manner. Heart rate-, BMI-, and HUNT-based nonexercise eCRF measures reported modest and comparable prognostic value. Given that these measures can be derived from simple, routinely collected clinical and lifestyle variables, eCRF may represent a practical and accessible approach for identifying older adults at elevated risk of CMM, particularly when direct fitness testing is not feasible. Further studies are needed to evaluate the potential utility of incorporating nonexercise eCRF measures into cardiometabolic risk assessment and preventive strategies and establish their role in routine clinical practice.

## Potential Competing Interests

The authors report no competing interests.

## Ethics Statement

English Longitudinal Study of Aging Wave 4 received ethical approval from the National Hospital for Neurology and Neurosurgery & Institute of Neurology Joint Research Ethics Committee on October 12, 2007 (07/H0716/48), and all participants provided written informed consent. Ethical approvals for the other waves in the ELSA project can be found on the website: https://www.elsa-project.ac.uk/ ethical-approval.

## Declaration of Generative AI and AI-Assisted Technologies in the Writing Process

During the preparation of this work the authors used 4o version of ChatGPT in order to refine writing and enhance clarity. After using this tool, the authors reviewed and edited the content as needed and take(s) full responsibility for the content of the publication.
