# Supplementary material for: Estimated Cardiorespiratory Fitness and Incident Cardiometabolic Multimorbidity in Older Adults: A Prospective Cohort Study
Source: Mayo Clin Proc Innov Qual Outcomes. 2026 Jul 27;10(5):100741. doi: 10.1016/j.mayocpiqo.2026.100741 (PMC13429898; doi:10.1016/j.mayocpiqo.2026.100741)
Supplement: Supplemental Materials 1-3 [file mmc1.pdf]

Supplementary Material S1. STROBE 2007 Statement—Checklist of items that should be included in reports of cohort studies

Section/Topic	Item #	Recommendation	Reported on page #
**Title and abstract**	1	(*a*) Indicate the study's design with a commonly used term in the title or the abstract	Page 1
		(*b*) Provide in the abstract an informative and balanced summary of what was done and what was found	Page 2
**Introduction**			
Background/rationale	2	Explain the scientific background and rationale for the investigation being reported	Page 4
Objectives	3	State specific objectives, including any prespecified hypotheses	Page 4
**Methods**			
Study design	4	Present key elements of study design early in the paper	Study population
Setting	5	Describe the setting, locations, and relevant dates, including periods of recruitment, exposure, follow-up, and data collection	Study population
Participants	6	(*a*) Give the eligibility criteria, and the sources and methods of selection of participants. Describe methods of follow-up	Study population
		(*b*) For matched studies, give matching criteria and number of exposed and unexposed	Not applicable
Variables	7	Clearly define all outcomes, exposures, predictors, potential confounders, and effect modifiers. Give diagnostic criteria, if applicable	Exposure, covariates and outcome
Data sources/ measurement	8*	For each variable of interest, give sources of data and details of methods of assessment (measurement). Describe comparability of assessment methods if there is more than one group	Exposure, covariates and outcome
Bias	9	Describe any efforts to address potential sources of bias	Statistical analyses
Study size	10	Explain how the study size was arrived at	Statistical analyses
Quantitative variables	11	Explain how quantitative variables were handled in the analyses. If applicable, describe which groupings were chosen and why	Statistical analyses
Statistical methods	12	(*a*) Describe all statistical methods, including those used to control for confounding	Statistical analyses
		(*b*) Describe any methods used to examine subgroups and interactions	Statistical analyses
		(*c*) Explain how missing data were addressed	Not applicable
		(*d*) If applicable, explain how loss to follow-up was addressed	Not applicable

		(*e*) Describe any sensitivity analyses	Statistical analyses
**Results**			
Participants	13*	(a) Report numbers of individuals at each stage of study—eg numbers potentially eligible, examined for eligibility, confirmed eligible, included in the study, completing follow-up, and analysed	Study population
		(b) Give reasons for non-participation at each stage	Study population
		(c) Consider use of a flow diagram	Study design and population
Descriptive data	14*	(a) Give characteristics of study participants (eg demographic, clinical, social) and information on exposures and potential confounders	Results; Table 1
		(b) Indicate number of participants with missing data for each variable of interest	
		(c) Summarise follow-up time (eg, average and total amount)	Results
Outcome data	15*	Report numbers of outcome events or summary measures over time	Results
Main results	16	(*a*) Give unadjusted estimates and, if applicable, confounder-adjusted estimates and their precision (eg, 95% confidence interval). Make clear which confounders were adjusted for and why they were included	Results; Figures 1-3
		(*b*) Report category boundaries when continuous variables were categorized	Results; Figures 1-3
		(*c*) If relevant, consider translating estimates of relative risk into absolute risk for a meaningful time period	
Other analyses	17	Report other analyses done—eg analyses of subgroups and interactions, and sensitivity analyses	Results; Supplementary Material S3
**Discussion**			
Key results	18	Summarise key results with reference to study objectives	Discussion
**Limitations**			
Interpretation	20	Give a cautious overall interpretation of results considering objectives, limitations, multiplicity of analyses, results from similar studies, and other relevant evidence	Discussion
Generalisability	21	Discuss the generalisability (external validity) of the study results	Discussion
**Other information**			
Funding	22	Give the source of funding and the role of the funders for the present study and, if applicable, for the original study on which the present article is based	Title page

Supplementary Material S2. Derivation of the analytic sample

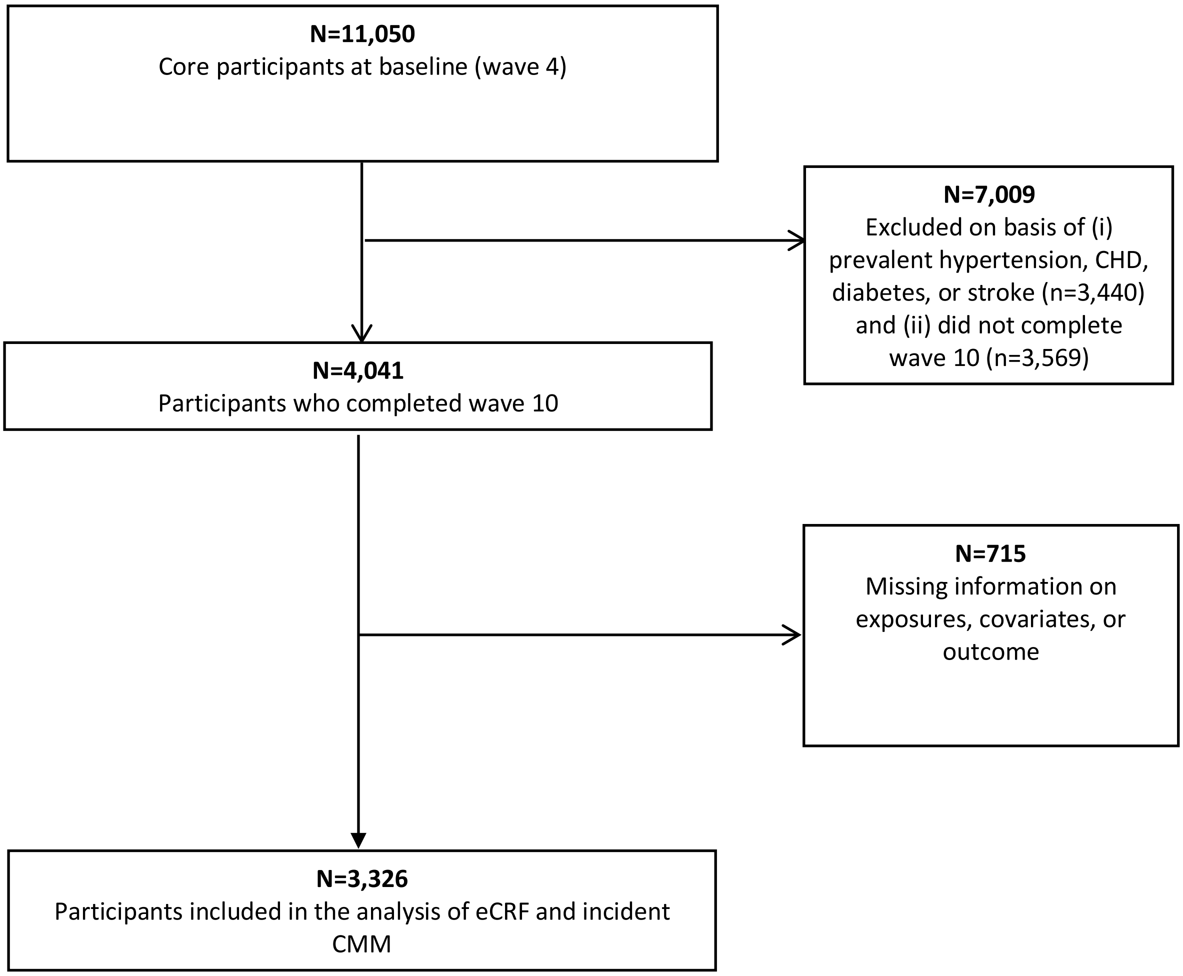

CMM, cardiometabolic multimorbidity; eCRF, estimated cardiorespiratory fitness

Supplementary Material S3. Associations of HUNT-based estimated cardiorespiratory fitness with cardiometabolic multimorbidity

Model 1

HUNT-based eCRF	Events / Participants	OR (95% CI)	*p*-value
Per MET increase	197 / 3326	0.66 (0.59, 0.73)	<.001
Tertile 1 (1.38-8.38)	92 / 1109 (8.3%)	ref	
Tertile 2 (8.39-9.79)	55 / 1109 (5.0%)	0.41 (0.28, 0.59)	<.001
Tertile 3 (≥9.80)	50 / 1108 (4.5%)	0.23 (0.15, 0.36)	<.001

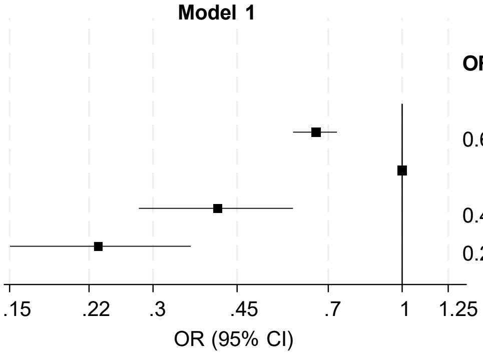

Model 2

HUNT-based eCRF	Events / Participants	OR (95% CI)	*p*-value
Per MET increase	197 / 3326	0.73 (0.65, 0.82)	<.001
Tertile 1 (1.38-8.38)	92 / 1109 (8.3%)	ref	
Tertile 2 (8.39-9.79)	55 / 1109 (5.0%)	0.53 (0.36, 0.78)	<.001
Tertile 3 (≥9.80)	50 / 1108 (4.5%)	0.36 (0.22, 0.58)	<.001

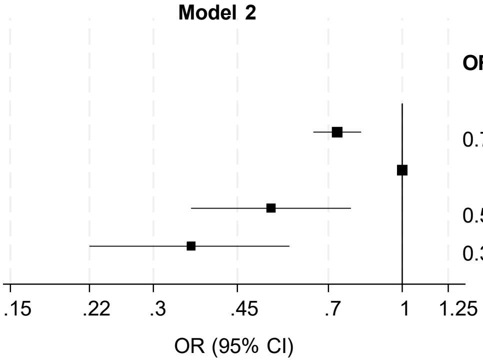

CI, confidence interval; HUNT, Nord-Trøndelag Health Study; OR, odds ratio; Values in brackets represent the absolute event proportions
Model 1: Adjusted for age and sex
Model 2: Model 1 plus alcohol consumption, systolic blood pressure, total cholesterol, high-density lipoprotein cholesterol, triglycerides, and handgrip strength
